# Photodynamic therapy for the precise treatment of localized prostate cancer

**DOI:** 10.3389/fonc.2025.1454392

**Published:** 2025-02-05

**Authors:** Youcheng Xu, Qinyuan Tan, Chong Sun, Yuefeng Jia, Shengxian Li, Xuecheng Yang

**Affiliations:** ^1^ Department of Urology, Affiliated Hospital of Qingdao University, Qingdao, China; ^2^ Department of Urology, The People’s Hospital of Jimo, Qingdao, China; ^3^ Department of Spine Surgery, Affiliated Hospital of Qingdao University, Qingdao, China

**Keywords:** photodynamic therapy, localized prostate cancer, photosensitizer, precise treatment, combined therapy

## Abstract

Over the past 20 years, early diagnosis of prostate cancer has become increasingly prevalent due to the promotion of prostate-specific antigens, and its treatment has become a focal point. However, there are some drawbacks associated with therapies for early prostate cancer, such as active surveillance and radical prostatectomy, which may include urinary incontinence, erectile dysfunction, and urinary tract infection. In contrast, photodynamic therapy (PDT) is introduced into the treatment of prostate cancer because of its advantages, such as high precision to tumor cells, low toxicity, and no radiation. Compared to radical prostatectomy, the PDT has low risk and minimal trauma. Although PDT is in the early stages of clinical development, it holds promise for the effective treatment of localized prostate cancer. Herein, we reviewed studies on the mechanisms of PDT and photosensitizers for prostate cancer. Given the rapid development of nanotechnology, photosensitizers wrapped by nanomaterials have emerged as new option with significant advantages, particularly of in achieving high tumor selectivity using functional nanomaterials. Numerous PDT clinical trials on prostate cancer have been conducted worldwide. We also reviewed the results of a few photosensitizers in these clinical trials. However, a few limitations and challenges regarding PDT for prostate cancer still exist. In addition, future development and potential clinical application strategies of future PDT are predicted.

## 1 Introduction

Prostate cancer is the second common malignant tumor in males (1). Over the past 20 years, there has been significant development in therapies for localized prostate cancer. In the past, traditional approaches for the treatment of localized prostate cancer mainly included active surveillance, radical prostatectomy (RP), and radiation therapy. However, RP can lead to some side effects, such as erectile dysfunction, urinary incontinence, and urinary tract infections (2). Active surveillance is another option. It may reduce overtreatment in some patients with early-stage prostate cancer, but the potential disadvantage of this approach is the psychological burden arising from delaying RP (3). Consequently, focal treatment for localized prostate cancer has gained researchers’ attention. This approach causes minimal damage to the surrounding tissues and preserves quality of life (4), because it destroys only the tumor or the area containing it (2). The current treatments for localized prostate cancer mainly include high-intensity focused ultrasound (HIFU) (4), cryotherapy (5), irreversible electroporation (6), and photodynamic therapy (PDT).

**Figure 1:**
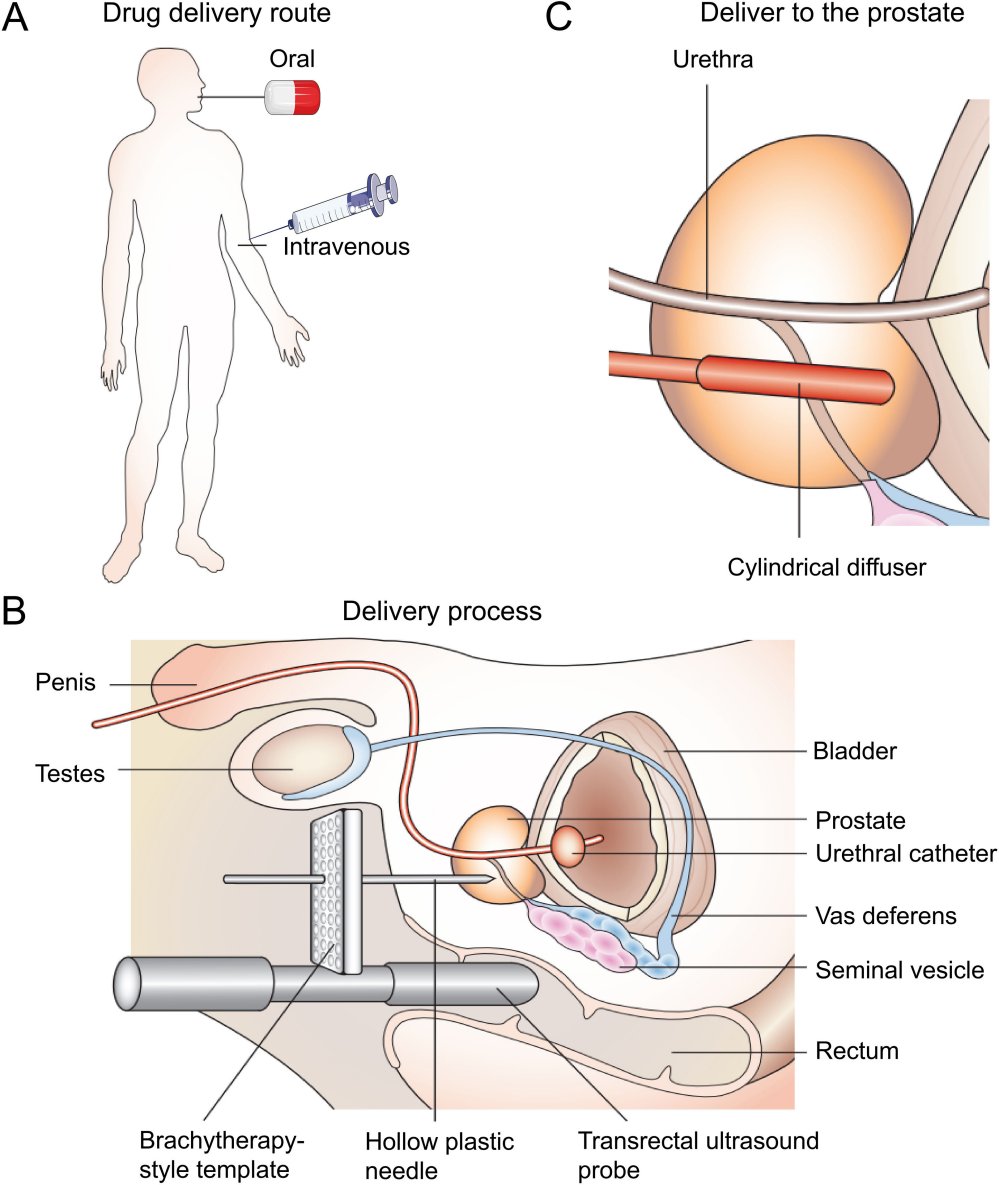
The use of PDT for prostate cancer. **(A)** The photosensitizers are administered either orally or intravenously. After a suitable drug–light interval, the drug is activated by a low-power laser light. **(B)** The light is most commonly delivered using a brachytherapy-style perineal template with transrectal ultrasound guidance. Hollow plastic needles are inserted into the prostate, and cylindrically diffusing fibers are positioned within the hollow needles. This part of the procedure is carried out under general anesthetic, with the patient in the lithotomy position. **(C)** The cylindrical diffuser fibers deliver light to the prostate at the appropriate drug–light interval (14).

PDT originated in the 1960s when Lipson et al. reported a porphyrin mixture (7). Schwartz then prepared and named it a hematoporphyrin derivative (HPD). In the 1970s, early preclinical and clinical studies expanded rapidly to explore the therapeutic potential of HPD (8–10). Since the approval of certain light applicators and photosensitizers (PSs), PDT has gained increasing attention. It mainly involves three components: light, tissue oxygen, and PSs (11). Compared with other focal therapies, PDT has lower toxicity and limited side effects, and does not utilize ionizing radiation (12). Furthermore, PDT equipment is cheaper and requires less room. PDT is a clinically approved and minimally invasive treatment for early-stage cancer (13). The photodynamic therapy procedures for prostate cancer is shown in **Figure 1** (14).

This article is an overall review of studies on PSs and PDT in localized prostate cancer. Moreover, it addresses current limitations and future perspectives.

## 2 The mechanisms of PDT

**Figure 2:**
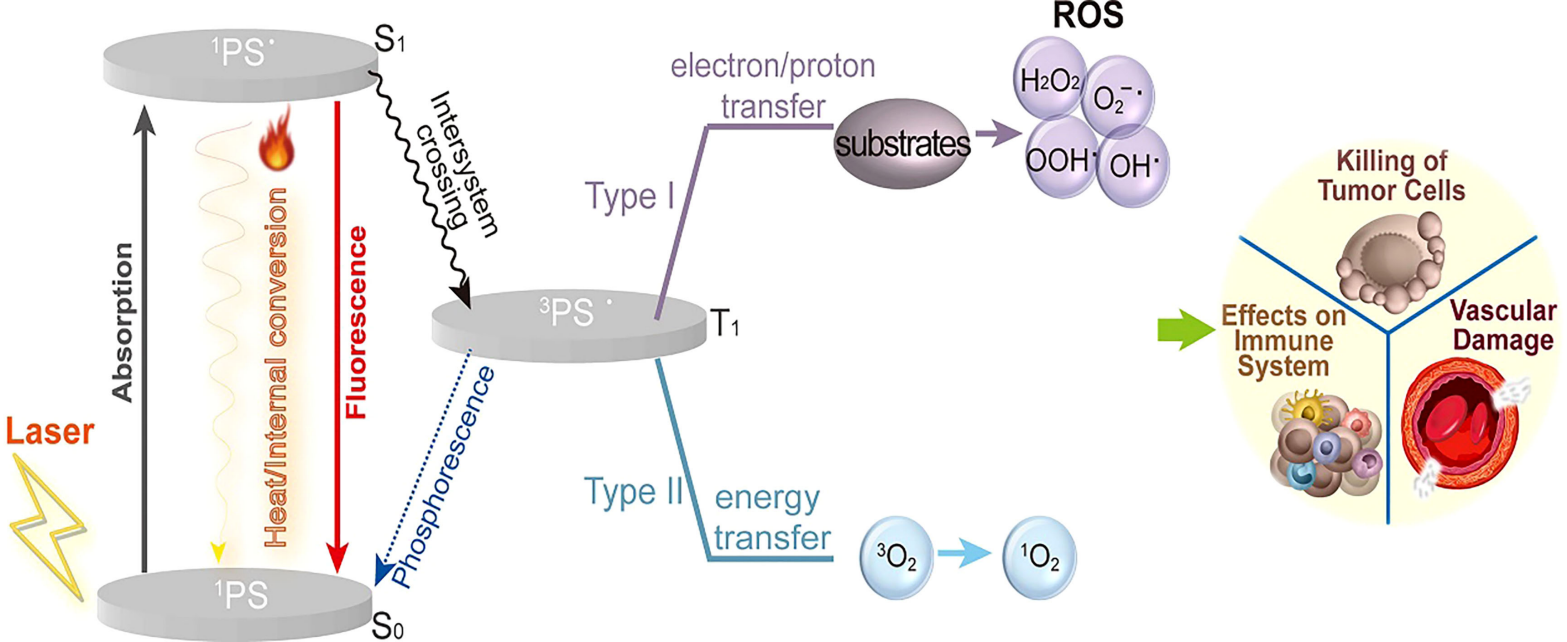
The mechanism of PDT. Light, oxygen, and photosensitizers are three primary elements of PDT required to eliminate tumors. The activated photosensitizers release energy by emitting light and heat or transforming into a triplet state. Then the triplet state PS produces reactive oxygen species (ROS) by two types of mechanisms. Ultimately, these processes lead to vascular damage, affecting the immune system and causing cell death (15).

The mechanisms of PDT are depicted in **Figure 2** (15). Light, oxygen, and photosensitizers are the three primary elements of PDT for tumor elimination (16). Photodynamic responses involve both photochemical and photophysical reactions. When PSs are exposed to light of a particular wavelength, they reach a higher-energy state called the singlet state. In this state, activated PSs release energy by emitting light, heat, or transforming into a triplet state (intermediate energy state) before returning to the ground state (11). Triplet-state PS can produce reactive oxygen species (ROS) via two mechanisms. In one of the mechanisms, PSs interact directly with substrates, transferring protons or electrons to produce organic radicals. Further reactions between cellular oxygen and these free radicals result in the generation of ROS, such as superoxide anions, peroxides, and hydroxyl radicals. The other mechanism involves the formation of singlet oxygen. When singlet oxygen interacts with biomolecules, its primary products undergo chain reactions related to free-radical peroxidation (17, 18). Ultimately, these processes lead to vascular damage, affecting the immune system and causing cell death (19).

There are three mechanisms that can explain the cytotoxicity of ROS in tumors. Firstly, ROS can lead to vascular disorders and thrombosis associated with tumors by destroying the vascular endothelial cells of the tumors. This means a reduction in the supply of oxygen and nutrients to the tumor. Secondly, ROS can cause an oxidative stress reaction, leading to the activation of the protein kinase pathway or the expression of cytokines and transcription factors. These reactions can lead to cell necrosis and apoptosis. Thirdly, ROS can induce necrosis and apoptosis of tumor cells, stimulating the activation of T cells and releasing numerous pro-inflammatory factors. Moreover, they induce acute inflammatory and anticancer immune responses (20, 21).

## 3 Basic research on PDT for the precise treatment of prostate cancer therapy

Light, oxygen, and PSs are the three primary elements of PDT for tumor elimination. Among them, the selection and application of PSs are the key factors affecting the efficacy of PDT. Since the 1950s, different kinds of photosensitizers have been discovered and tried for the treatment of cancer (15). They are divided into three generations according to the time of discovery. The characteristics of different PSs are listed in **Table 1**.

**Table 1:** The characteristics of different photosensitizers.

PSs	Activation wavelength	Maximum wavelength	Advantages	Disadvantages	Reference
Talaporfin	450 nm	660 nm	Short drug-light interval; selectivity for cancer cells	Visual disturbances reported	(22)
Temoporfin	510 nm	690 nm	Selectivity for cancer cells	Visual disturbances reported	(23)
Verteporfin	530 nm	692 nm	Selectivity for cancer cells; short drug-light interval (15-30 min)	Less tissue penetration	(24)
Hematoporph	405 nm	635 nm	Preparation less heterogenous than HpD derivatives	Prolonged skin photosensitivity; long drug-light intervals; suboptimal tumor selectivity	(25)
5-aminolevulinic acid (5-ALA)	410 nm	635 nm	Selectivity for cancer cells; short drug-light interval (up to 4 h)	Less tissue penetration	(26)
Motexafin lutetium	432 nm	732 nm	Short drug-light interval (3 h); no reported skin photosensitivity	No disadvantages reported	(27)
Hypericin	400 nm	595 nm	Longer absorption wavelengths metabolized rapidly, decreasing the side effects.	Poor tumor targeting capacity and low permeability of local delivery.	(28)
Phthalocyanine	480 nm	680 nm	Longer absorption wavelengths metabolized rapidly, decreasing the side effects.	Poor tumor targeting capacity and low permeability of local delivery.	(29)
Methyl aminolevulinic acid	450 nm	638 nm	Selectivity for cancer cells; short drug-light interval (up to 4 h)	Less tissue penetration	(30)
Pyropheophorbide-a	425 nm	634 nm	Preparation less heterogenous than HpD derivatives	Less tissue penetration	(31)
Polycyclic quinone	492 nm	560 nm	Short drug-light interval (3 h); no reported skin photosensitivity	No disadvantages reported	(32)

### 3.1 First-generation PSs

In the 1950s, hematoporphyrin was found to accumulate in tumor tissues and induce cell death after exposure to light (33). The observed phenomenon indicated that the photodynamic effect of hematoporphyrin resulted in cell death, involving the production of toxic byproducts and ROS (25). In the 1970s, an HPD was used as a PS in PTD to treat bladder cancer (34). By 1978, the successful application of first-generation PSs in treating different tumors was reported by Dougherty et al (10). In 1985, the photoinduced toxicity of an HPD used on rat prostate cancer cells was studied (25). The occurrence of prostate tissue necrosis during PDT depends on the PS content in the prostate. Although the distribution of porphyrin in the prostate and other urological organs has been investigated in mice, there are significant discrepancies between the mouse model and humans. Physiological and morphological similarities exist only between the prostates of humans and other primates. Therefore, primates are preferred for assessing porphyrin distribution in the prostate and other organs. In addition, the primate prostate contains a high concentration of zinc (35), making it a suitable choice to study zinc-containing metalloporphyrins in prostate cancer treatment (36). Although zinc accumulation is high in prostatic tissues, preferential localization to the two lobes of the primate prostate has not been achieved. Interpreting this result is challenging because of limited data on the effects of zinc on prostatic physiology. There are three possible explanations for this finding: (1) Zinc may be preferentially taken up by prostatic epithelial cells, especially when the zinc atoms are placed inside the porphyrin molecule rather than at the periphery. (2) The zinc characteristics of the metalloporphyrin complexes may have disappeared. (3) Zinc accumulation is under the androgenic control of the prostate (37). Even if zinc-HPD is preferentially taken up by prostatic tissues, it is clear from the spectrum that the most useful absorption peak at 630 nm is lost. There exist a few other first-generation photosensitizers, such as verteporfin (24) and talaporfin (22).

However, numerous shortcomings limit the application of HPD. For example, the low absorption rate of light, owing to the short excitation wavelength, makes it difficult for light to penetrate deeper tissues and effectively execute PDT. In addition, it is difficult to synthesize and purify HPDs. Lastly, HPDs can easily induce phototoxicity due to their long half-lives.

### 3.2 Second-generation PSs

Second-generation PSs include phthalocyanines, phenothiazines, polycyclic quinones (32), and porphyrin derivatives. Compared with first-generation PSs, second-generation PSs have many improvements. First, they can be activated by near-infrared (NIR) light due to their longer absorption wavelengths. The ROS rate is related to the effect depth of these PSs. In addition, second-generation PSs can be rapidly metabolized, thereby reducing their side effects (38, 39).

Porphyrin-derived PSs primarily contain endogenous and exogenous porphyrins. Among endogenous porphyrins, 5-aminolevulinic acid (5-ALA) is widely used as a precursor for heme synthesis (26). It is an endogenous biochemical substance that is subjected to ALA anhydrase and a series of enzymatic actions to produce protoporphyrin IX with strong photosensitizing effect (40). In 1990, Kennedy et al. first used this technique for tumor ablation (41). Later, Nakayama et al. used ALA-PDT to treat prostate cancer cells and found that it led to rapid ROS-induced cell death and the suppression of cell proliferation through several pathways (42). However, 5-ALA is easy to decompose if it is in direct sunlight and its penetration is low. 5-ALA is not the optimal for refractory and thick tumors. Methotrexate was combined with ALA because it could cause an increase in protoporphyrin IX, which overcame disadvantages of 5-ALA (30). Exogenous porphyrins, such as chlorin e6 (Ce6) and photochlor (HPPH), are comprehensively used in PDT (43). HPPH, with its long excitation wavelength, is used for solid tumors. Furthermore, its low phototoxicity eliminates the need to avoid light exposure after injection. Ce6 (44), synthesized from pyropheophorbide-a, is suitable for PDT because it can efficiently produce singlet oxygen. However, Ce6 has a hydrophobic nature, making its application difficult. Using HPPH for treatment presents a significant limitation: the non-specific uptake and extensive diffusion of HPPH can weaken its effectiveness and result in off-target effects. Researchers try to revise the physical structure of HPPH, which can load it into new drug delivery systems. This will be mentioned in discussing the application of third-generation photosensitizers.

Polycyclic quinone, a natural PS, is widely found in plants. It can generate free radicals, singlet oxygen, and superoxide anions when used in PDT, which suggests that it can trigger two types of photosensitive reactions (45). Representative polycyclic compounds include hypericin, hypocrellin, and curcumin. Hypericin extracted from Hypericum perforatum L. has excellent photosensitivity to light (28). It can rapidly induce the release of Ca^2+^ from the endoplasmic reticulum, a process possibly correlated with cell apoptosis. In addition, the light-independent effects of hypericin, including the ultrastructure of organelles, protein synthesis, and ROS generation, have been reported by Huntosova and Stroffekova (46). Hypocrellin occurs as two types: HA and HB; both exhibit high ROS generation ability and rapid clearance. Therefore, hypocrellin is a promising anticancer PS for PDT. Curcumin is well known for its photosensitivity and anticancer properties. These properties exert synergistic effects when used in PDT.

Phenothiazine is a widely used PS. Methylene blue (MB), new methylene blue, dimethyl methylene blue (DMMB), and toluidine blue O are commonly used in this research. A previous study revealed that DMMB may act through the cellular stress axis to induce cell death. MB triggers cellular responses such as necrosis, apoptosis, and autophagy, relying on the nonspecific generation of many oxidant species (47).

Phthalocyanine is a porphyrin analog that can modify cores and substituents, incorporating zinc, silicon, or aluminum to optimize its properties (48). Consequently, they can be activated by light in the wavelength range of 750–900 nm. In addition, phthalocyanine PSs have ideal characteristics, such as low phototoxicity, quick clearance, and high fluorescence signal generation (29). Therefore, they can be used in photodiagnosis and photodynamic treatments.

However, second-generation PSs still have some disadvantages, such as poor tumor-targeting capacity and low permeability for local delivery.

### 3.3 Third-generation PSs

**Figure 3:**
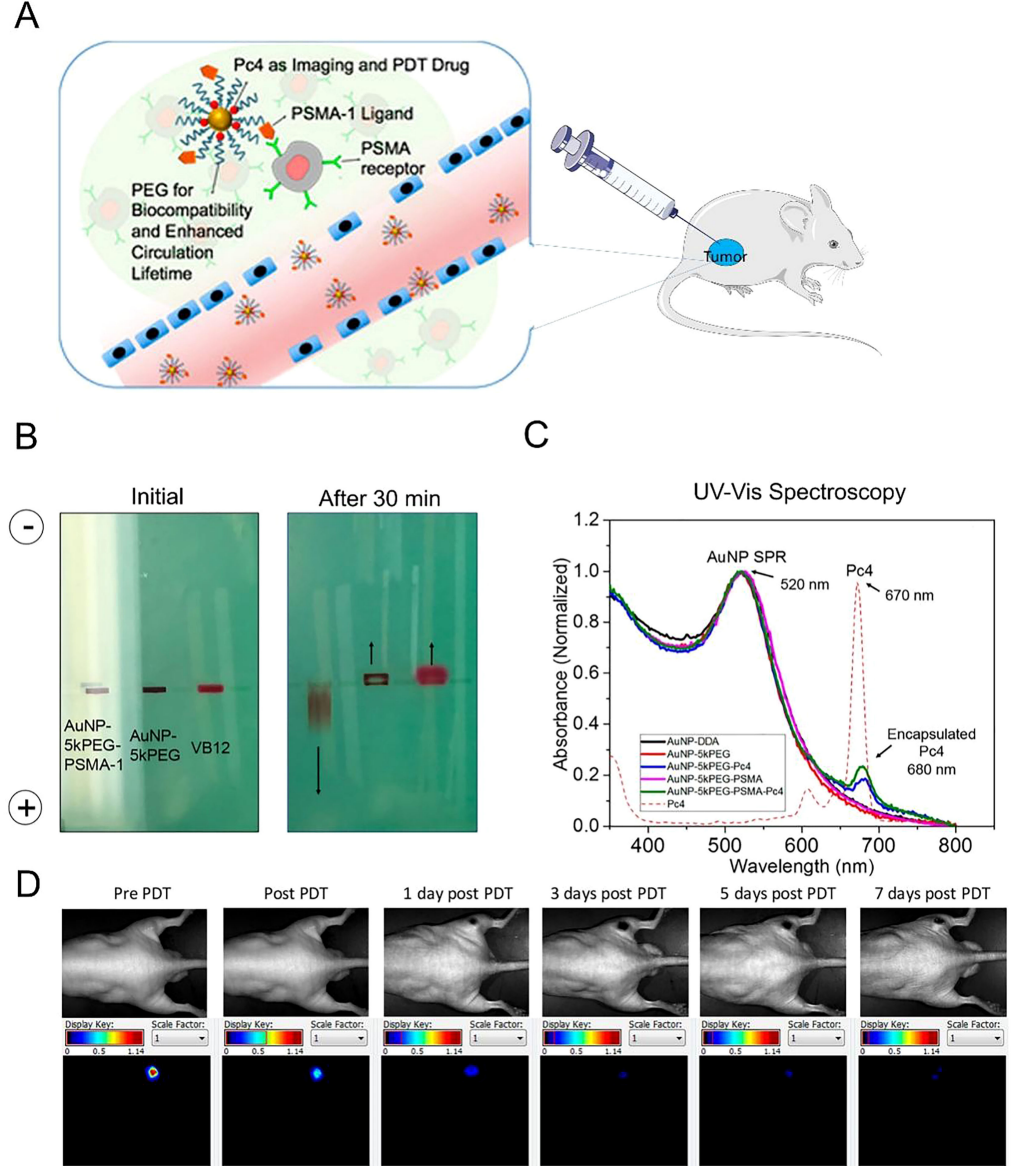
Basic research on photosensitizers in prostate cancer therapy. **(A)** PDT was administered to the mice. **(B)** After rejection, the initial electrophoresis variations and those after 30 min. **(C)** The different absorbances of AuNPs SPR and encapsulated Pc4 at different wavelengths. **(D)** Imaging and fluorescent labeling after rejection (51).

The third-generation PSs are based on second-generation PSs and combined with substances with biological properties to improve the targeting of photodynamic therapy and they can attain high tumor selectivity by using functional nanomaterials (49) or conjugating target molecules. DR_2_ is a bimolecular conjugate composed of an anti-androgen molecule that can release NO under the influence of light and a photosensitizer (pheophorbide). DR_2_ can modulate the NF-KB/YY1/RKIP loop, which, in turn, controls the growth and apoptosis of prostate cancer cells (50). The basic method of research on PSs for prostate cancer is depicted in **Figure 3** (31). Prostate-specific membrane antigen (PSMA) is a glycoprotein commonly found on the surface of prostate cancer cells and has attracted considerable attention as a target for delivery. Pyropheophorbide-a is a PS that lacks specificity for cancer cells (52). However, inhibitors known as phosphoramidate peptidomimetic PSMA are used for both intracellular delivery and cell-surface labeling of prostate cancer cells (53). Thus, a conjugation of the PSMA inhibitor to pyropheophorbide-a has been analyzed (54, 55). Deks et al. effectively improved the accuracy and efficacy of surgical treatment of prostate cancer by combining fluorescence-guided surgery for PSMA with tumor-targeted PDT (56).

Various functional nanomaterials have emerged owing to the rapid development of nanotechnology (57). These materials are carriers for delivering PSs with low water solubility (58). In addition, certain nanomaterials can be used directly as PSs (59). Examples include gold nanoparticles (60), silica nanoparticles (61), magnetic nanoparticles (62), polymeric nanoparticles (63), liposomes (64), and quantum dots (65).

In recent years, gold nanoparticles have been shown to have unique chemical and physical properties, including fluorescence enhancement and surface plasmon resonance, making them suitable for drug delivery and targeting (66). PSMA-targeted nanoparticles complex is a new drug delivery system. By combining nanoparticles with PSMA-targeted ligands, it is possible to improve the targeting and bioavailability of drugs in prostate tissue. PSMA-targeted gold nanoparticles have been synthesized and can effectively deliver drugs to targeted cells. The animals treated with these nanoparticles showed tumor remission after 14 days, indicating their potential for therapeutic intervention (31). Luo et al. bound PSMA-targeting ligands and gadolinium (Gd III) complexes to the gold nanoparticles surface. They are used in magnetic resonance (MR)-guided prostate cancer-targeted therapy. The results showed that the binding of gold to Gd (III) inhibited prostate cancer more effectively after radiotherapy (67). Titanium dioxide (TiO_2_) has been found to be photoactive upon irradiation. It also has high stability, appropriate biocompatibility, and low toxicity and is used in anticancer PDT (68). However, only UV light can activate pure TiO_2_, and its tissue penetration is low, limiting its application in PDT (69). Therefore, researchers have attempted to use targeted TiO_2_ nanoparticles in PDT. The results showed that TiO_2_ decreased the viability of cancer cells (61). Silica has a tetrahedral structure. According to pore diameter, the silica nanoparticles are classified as macropores (>50 nm), mesopores (2–50 nm), and micropores (<2 nm) (70). Mesoporous silica nanoparticles (MSNs) are considered suitable PS carriers because of their excellent biocompatibility and large surface area. As biocompatible materials, MSNs can be decomposed into water-soluble and non-toxic orthosilicic acid (Si (OH)_4_) in a physiological environment and then extracted in urine (70, 71). Magnetic nanoparticles (MNPs) have unique biocompatibility, stability, and physical properties (72). It is known that a permanent magnet can generate an external magnetic field with controlled interactions with MNPs. These MNPs can be used to study the specific location of a tumor within an organism and release drugs, thus creating a targeted drug delivery system. PSMA-targeted MNPs were developed by Ngen et al. After intravenous administration of 50 mg/kg of MNPs in a mouse model, evaluation using T2-weighted MRI determined the concentration required for therapy. Compared to PSMA (-) tumors, the nanoparticles initially aggregated at the tumor periphery, and contrast enhancement was observed in PSMA (+) tumors after administration (73). Although the U.S. Food and Drug Administration (FDA) has approved some MNPs, many of them cause side effects, such as damage to the brain, liver, skin, and nervous system. Therefore, their use should be controlled using organic or inorganic compounds (74). In summary, the researches of targeting PS in prostate cancer are listed in **Table 2**.

**Table 2:** The research of targeting PS in prostate cancer.

Targeting PS	Research	Result
Gold nanoparticle	Luo et al. bound PSMA-targeting ligands and gadolinium (Gd III) complexes to the gold nanoparticles surface.	The binding of gold to Gd (III) inhibited prostate cancer more effectively after radiotherapy
Titanium dioxide	Researchers have attempted to use targeted TiO_2_ nanoparticles in PDT.	The results showed that TiO_2_ decreased the viability of cancer cells
Magnetic nanoparticles (MNPs)	PSMA-targeted MNPs were developed by Ngen et al. have unique biocompatibility, stability, and physical properties	Their use should be controlled using organic or inorganic compounds
Polymeric nanoparticles	Chen et al. developed polymeric NCs that encapsulated a combination of quercetin and doxorubicin for active targeting of prostate cancer	Polymeric NCs with well-defined covalently stabilized structures have been generated as potentially safe and universal therapeutic nanocarriers
Liposomes	The co-delivery of resveratrol (Res) and docetaxel (Doc) via liposomes was introduced by Zhang et al. Lipid carriers are easier to prepare than other nanocarriers and are non-toxic.	Prolonged survival compared to the controls exposed to Res/Doc without liposomes
Quantum dots	A nanosystem with graphene oxide for intravenous therapy was developed by Jiang et al.	In *in vitro* and *in vivo* studies, this carrier showed an ability to target prostate cancer

In addition, new nanoparticles have been used in PDT for prostate cancer treatment (75). Polymeric nanoparticles are prepared from synthetic or natural polymers with diameters less than 1 µm. These polymeric nanoparticles are further classified as nanocapsules (NCs) or nanospheres (NSs) (76, 77). NCs consist of a solid/liquid nucleus with a shell, with the drug dissolved in the core, although some can be on the surface of the NCS. In contrast, NSs consist of a solid matrix without a polymer shell. The drug can then be absorbed into the matrix (78). Chen et al. developed polymeric NCs that encapsulated a combination of quercetin and doxorubicin for active targeting of prostate cancer. Polymeric NCs with well-defined covalently stabilized structures have been generated as potentially safe and universal therapeutic nanocarriers (79). Since liposomes were first described by Bangham, active research has been conducted in this field (80). Liposome self-assembly and multifunctional carrier material containing lipid bilayers with cholesterol or phospholipids and hydrophobic drugs can bind with the lipid bilayers, as hydrophilic drugs are encapsulated in the internal water chambers (81). Lipid carriers are easier to prepare than other nanocarriers and are non-toxic (82, 83). The co-delivery of resveratrol (Res) and docetaxel (Doc) via liposomes was introduced by Zhang et al. Animal experiments showed that the drugs could be delivered to prostate PC-3 cells. Mice treated with Res/Doc-containing liposomes exhibited prolonged survival compared to the controls exposed to Res/Doc without liposomes (84). Quantum dots with diameters of 2−10 nm are composed of semiconductor elements with unique electronic and photoluminescent properties (85). The core of semiconductor materials, such as lead selenide or cadmium selenide, determines their optical properties (86–88). In castration-resistant prostate cancer (CRPC), a nanosystem with graphene oxide for intravenous therapy was developed by Jiang et al. The graphene quantum dots were first cross-linked by disulfide bonds and then formed by approximately 200 nm derivatives that could load enzalutamide. In *in vitro* and *in vivo* studies, this carrier showed an ability to target prostate cancer, which could be internalized by CRPC cells, inhibit the proliferation of cancer cells, and reduce the side effects of drugs *in vivo (* 23). Taken together, nanomaterials are gaining attention in PDT for prostate cancer due to their numerous advantages (75, 89, 90). Besides, there are also some other manners of using nanomaterials to the cancer therapy. Researchers reported that they designed a new carrier loaded glucose oxidase and disulfiram prodrug, and the carrier used copper (II)-based metal–organic framework, which could be combined with Cu^2+^ (27) was used to enhance tumor-specific therapy (91).

As we all know, generating cytotoxic singlet oxygen for prostate cancer treatment depends on the ability that PS transfers energy from lasers to dissolved oxygen (92, 93). However, in tumors the oxygen supply is not adequate, which will impair the effectiveness of PDT. Hypoxia is common because of the reduced oxygen supply by deteriorated diffusion and disturbed microcirculation in prostate cancer, which are also the unique characteristics and antitumor mechanisms of prostate cancer. In addition, PDT worsens hypoxia through vascular shutdown effects and oxygen consumption. Low oxygen can reduce photodynamic efficacy about PS, preventing PDT from achieving full potential of cancer therapeutic. To ensure PDT efficacy, some traditional experiments have tried to optimize tumor oxygenation. For example, extending irradiation with low fluence rate and dividing irradiation into dark-light circles have both been investigated for better tumor reoxygenation. To actively increase the oxygen of tumor, hyperbaric oxygen inhalation has been used. However, there exist potential toxic effects about excessive oxygen, an impediment to its clinical use. Therefore, it is important for photodynamic therapy to optimize the efficacy with limited oxygen. To overcome this challenge, researchers load PS into perfluorocarbon nanodroplets, developing novel oxygen self-enriched photodynamic therapy. At the given oxygen partial pressure, perfluorocarbon can maintain a high oxygen content because of its high oxygen capacity. In their study, they demonstrated a platform called Oxy-PDT to achieve enhance efficacy (94). The PDT efficacy is determined by ^1^O_2_ generation rate because it is ^1^O_2_ for PDT to kill the cancer cells. In the Oxy-PDT agent, both oxygen and PS are enriched in the nanodroplet. Oxy-PDT can realize higher efficacy in hypoxic conditions and is considered the first PDT design. We anticipate its wide clinical application in the future.

We can discover that some of the third generation PSs are enhanced by some materials in therapy of prostate cancer compared to the first generation PSs. For example, Mesquita et al. demonstrated significant therapeutic potential by synthesizing fluorinated porphyrin derivatives and optimizing their application using nanotechnology (95).

## 4 Clinical research on PDT for prostate cancer

A great number of PDT clinical trials to treat prostate cancer have been performed worldwide. The first clinical study on PDT to treat prostate cancer was reported in *The Lancet* in 1990 (96).

As mentioned earlier, 5-ALA produces PpIX, which has strong photosensitivity and is activated at 420−460 nm. Clinical research in Germany has reported the efficacy of PpIX in treating prostate cancer. Adam et al. reported the use of ALA to identify positive surgical margins in endoscopic extraperitoneal and open retropubic RP, which could improve RP outcomes. Thirty-nine patients with prostate cancer received oral ALA and underwent RP (15 underwent open retroperitoneal RP and 24 underwent endoscopic extraperitoneal RP). The results showed that the sensitivity of the open RP group was significantly lower than that of the endoscopic RP group. As ALA-PDD/PTT (photodynamic diagnosis and photothermal therapy) can reduce the interval between drug administration and irradiation to 4 hours, the authors considered ALA-PDD during RP to be an effective method (97).

Many physicians have explored the treatment of prostate cancer using temoporfin activated at 652 nm (98). In 2002, Nathan et al. reported their phase I study involving 14 patients with prostate cancer experiencing local recurrence after radiotherapy. These patients were administered temoporfin-PDT, and the interval from intravenous injection to irradiation was 72 hours. After PDT, the prostate specific antigen (PSA) levels of nine patients decreased, and no tumors were found in the biopsies of five patients. Unfortunately, all patients underwent androgen deprivation therapy (ADT) as their PSA levels eventually increased. The researchers attributed this to partial gland coverage during irradiation (99). In 2006 (98), doctors in London reported another study involving temoporfin-PDT, wherein six patients with prostate cancer underwent focal PDT, resulting in a 48.3% decrease in PSA levels. Biopsy results revealed that all patients who underwent PDT had residual cancer. Among them, two patients underwent cryotherapy and brachytherapy, while three patients underwent external beam radiotherapy. Only one patient remained cancer-free 6 years after PDT. It is important to note that these trials were limited to areas detected by biopsy (100). The efficacy of temoporfin can be enhanced with improvements in light dosimetry and the accuracy of needle placement.

Motexafin lutetium is a potent PS, activated at 730−770 nm (101). It has been approved by the FDA for the treatment of malignant melanomas and breast cancer (102). In 2006, a phase I study of 16 patients with recurrent prostate cancer was reported by Du et al. (103). This study was the first to assess the PSA value, ranging from day one to several weeks after PDT. The results indicated that PSA levels significantly increased shortly after PDT, suggesting that cellular damage induced by PDT may result in a transient increase in PSA levels. Consistent with the results of other trials, the PSA value dropped below baseline within 2 months after PDT and then increased again (101).

At 763 nm NIR light can active vascular-targeted PSs called Tookad^®^, destroying tumor blood vessels and effectively killing cancer cells. This approach is called vascular-targeted photodynamic therapy (VTP). VTP is administered using a photosensitizer that is delivered intravenously and locally activated by laser fibers that emit a specific wavelength of light. Once activated, photosensitizers produce free radicals that cause severe forms of uniform local vascular damage within tissues, leading to controlled, non-thermal forms of coagulated tissue necrosis. Padeliporfin (WST11, Tookad^®^) is hydrophilic, whereas Padoporfin (WST09, Tookad^®^) is hydrophobic. WST11 is a new negatively charged, water-soluble palladium-bacterial chlorophyll derivative that has been used in vascular-targeted photodynamic therapy (VTP). *In vitro* results suggest that WST11 cell uptake, clearance, and phototoxicity are mediated by serum albumin trafficking. The first phase I/II trial of VTP in prostate cancer patients after external beam radiotherapy was conducted by Trachtenberg et al (104). Only two fibers were placed in the prostate to demonstrate safety in the first trial (105). Subsequently, a phase II trial was conducted. In this trial, 28 patients received WST09 (2 mg/kg). The results indicated a notable therapeutic effect with an appropriate light intensity and a safe drug concentration (106). In France, a phase II trial reported that 56 patients with prostate cancer (Gleason score ≤3 + 3) underwent VTP. After treatment for 6 months, the mean PSA value was 3.7 ng/mL, and there was no residual tumor in the targeted area (107). To determine the WST11 concentration, 40 patients with prostate cancer (Gleason score ≤3 + 3) received 2, 4, or 6 mg/kg of WST11. The results showed that 4 mg/kg of WST11 was the optimal dose (108). A Latin American trial (PCM304) evaluated the efficacy of PDT in men with prostate cancer. Twelve months after VTP, 60 (74%) patients had negative biopsy results (109). In 2019, a phase II trial assessed tumor control in 68 patients after VTP. The results were similar to those of earlier phase II studies; a quarter of the patients had positive biopsy results after 3 years. Without removing or destroying the prostate, it only causes short-term urinary incontinence and erectile problems and can be recovered within three months (110). Although VTP has many advantages, such as easy metabolism, lack of obvious side effects, and no drug-light interval, it must be used carefully in low-risk patients (111). The initial experience of patients who underwent VTP for unilateral low-risk prostate cancer was reported in a real-world study conducted in Germany. The researchers compared the short-term oncological outcomes in patients undergoing continuous RP. The biopsy results indicated that at 12 and 24 months after PDT, 27% of patients had low-and intermediate-risk prostate cancer, respectively. This study suggests that the complication rate of VTP is lower than that of RP. However, after VTP, recurrence and progression are common; therefore, a rigorous surveillance strategy is required (112). In addition, salvage RP after VTP seems feasible because it does not involve thermal ablation. Above all, the clinical researches on PDT for prostate cancer are listed in **Table 3**.

**Table 3:** Clinical research on PDT for prostate cancer.

Clinical trials	The process	The result	Reference
Adam et al. reported the use of ALA to identify positive surgical margins in endoscopic extraperitoneal and open retropubic RP, which could improve RP outcomes.	Thirty-nine patients with prostate cancer received oral ALA and underwent RP (15 underwent open retroperitoneal RP and 24 underwent endoscopic extraperitoneal RP).	The results showed that the sensitivity of the open RP group was significantly lower than that of the endoscopic RP group.	(97)
In 2002, Nathan et al. reported their phase I study involving 14 patients with prostate cancer experiencing local recurrence after radiotherapy.	These patients were administered temoporfin-PDT, and the interval from intravenous injection to irradiation was 72 hours.	After PDT, the prostate specific antigen (PSA) levels of nine patients decreased, and no tumors were found in the biopsies of five patients.	(99)
In 2006, a phase I study of 16 patients with recurrent prostate cancer was reported by Du et al.	This study was the first to assess the PSA value, ranging from day one to several weeks after PDT.	The results indicated that PSA levels significantly increased shortly after PDT, suggesting that cellular damage induced by PDT may result in a transient increase in PSA levels.	(103)
The first phase I/II trial of VTP in prostate cancer patients after external beam radiotherapy was conducted by Trachtenberg et al	Only two fibers were placed in the prostate to demonstrate safety in the first trial. Subsequently, a phase II trial was conducted. In this trial, 28 patients received WST09 (2 mg/kg).	The results indicated a notable therapeutic effect with an appropriate light intensity and a safe drug concentration	(104)
In France, a phase II trial	56 patients with prostate cancer (Gleason score ≤3 + 3) underwent VTP	After treatment for 6 months, the mean PSA value was 3.7 ng/mL, and there was no residual tumor in the targeted area	(107)

## 5 Current limitations and future perspectives of PDT

There are several limitations to use PDT for prostate cancer. These limitations differ between focal and whole-gland therapies.

The challenges with using focal therapy include the initial and future aspects. The former involves accurately predicting the behavior of prostate cancer, identifying cancer within the prostate, and treating the identified target volume. The latter involves ascertaining whether the intended treatment is administered at the planned treatment volume and ensuring the untreated part of the prostate is handled appropriately. In the PDT of prostate cancer using a PS linked to a monoclonal antibody, there is heterogeneity in antigen expression within a tumor or among different regions in the same prostate cancer. Multiple targeting devices can resolve this problem; however, their efficacy should be analyzed in preclinical studies.

There are some limitations to PDT of the whole gland. An adequate dose of the drug, oxygen, and light should be delivered to the entire gland to reliably achieve the whole-gland effect of PDT. When the effect does not extend beyond the gland, excess light or drugs do not matter. Future studies should explore the correlation between drug, oxygen, and light measurements, and PDT outcomes. The lack of an appropriate follow-up protocol for PDT of the entire gland remains a challenge. Although MRI is useful for identifying avascular lesions, the correlation between these lesions and long-term clinical outcomes and biopsy materials is not available (113). Whole-gland PDT has demonstrated significant morbidity after radiotherapy compared to that with other salvage treatments. For example, in a study assessing temoporfin, a rectourethral fistula was observed after a PDT rectal biopsy (99). Another study assessing padoporfin levels after radiotherapy reported two rectourethral fistulas (106). Whole-gland treatment has mainly been studied in patients who received radiotherapy before whole-gland PDT. Some patients may experience urinary dysfunction after whole-gland PDT. On the contrary, PDT for focal prostate cancer can identify accurately the tumor within the gland.

Several studies in canine and human prostates have demonstrated the ability of different PSs to induce necrosis in prostate cancer cells or prostate tissue. However, some PSs, such as porfimer sodium (Photofrin), induce toxicities, such as skin photosensitization (96). After using such PSs, it is necessary to avoid bright light for several days, thereby reducing the quality of life and introducing additional problems. For example, patients should stay in a room with reduced lighting for 3 days after using meso-tetra-hydroxyphenyl-chlorin to prevent skin photosensitivity (99).

The future holds the promise of detecting more prostate cancers at an early stage, thanks to the development of early detection techniques such as liquid biopsy. This implies that minimally invasive treatments, such as PDT, will benefit an increasing number of patients with prostate cancer. Additionally, there are treatment options available for locally recurrent prostate cancer and CRPC. This vast potential opens avenues for patients with prostate cancer, paving the way for more effective therapies. Studying the optical characteristics of different types of prostate cancer can help design a clinical reference strategy. The development of molecular targeting techniques has made PDT highly selective for tumors, with lower toxicity to surrounding tissues. While PDT is in its early stages compared to other ablative modalities, such as cryotherapy and HIFU, it has the suitable characteristics required for focal treatment in prostate cancer. In the future, PDT warrants further exploration in clinical trials. As mentioned above, the use of advanced nanoparticles in the third generation can overcome the known limitation of low penetration in PDT. Future objectives should focus on addressing biodegradability as the primary concern, enhancing the utilization of PDT for prostate cancer. Additionally, ongoing developments, especially in photosensitizer delivery and real-time feedback systems, make PDT an important addition to the range of treatment options under investigation for organ-confined prostate cancer (114). Development of new therapeutic diagnostics based on the combination of PDT and different imaging techniques to achieve treatment and diagnosis. This combination can help address poor biodistribution and selectivity through regional imaging, while therapeutics enable effective personalized treatment. Biomarkers can identify patients who are sensitive to PDT, allowing for personalized treatment. Besides, biomarkers such as photosensitizer-specific expression levels, tumor hypoxia, and immune status can predict PDT response.

Although PDT has these limitations, we can use it with other therapies. For example, PDT can combine the photothermal therapy (PTT) to enhance selectivity of cancer therapy (115).

## 6 Conclusion

The traditional treatments mainly include RP and active surveillance. However, RP can lead to some side effects, such as erectile dysfunction, urinary incontinence, and urinary tract infections. And the potential disadvantage of active surveillance is the psychological burden arising from delaying RP. Herein, we reviewed PDT for localized prostate cancer and noted that it is a minimally invasive treatment for localized prostate cancer and can preserve the functions of the surrounding organs, such as rectum, bladder, and neurovascular bundle. We also reviewed different PSs, spanning from the first to the third generations. The third generation is important because it is extensively used in PDT for prostate cancer, where an increasing number of new nanoparticles is being combined with PSs. This integration is expected to enhance the accuracy and safety of PDT for localized prostate cancer. Moreover, the promotion of prostate-specific antigens can facilitate early diagnosis of prostate cancer. It is noteworthy that we gathered not only studies on PSs but also those on PDT. An important point is that PDT can be repeated without cumulative toxicity. Therefore, if recurrent or residual diseases are identified, PDT can be used to ablate the remaining prostate tissue after focal therapy. Although there are limitations to PDT for localized prostate cancer, we anticipate that in the future, PDT will overcome these limitations and advance to the next level. We believe that PDT will undergo comprehensive development leading to precise application for the treatment of localized prostate cancer.
